# *In Vitro* efficacy of antimicrobial extracts against the atypical ruminant pathogen *Mycoplasma mycoides* subsp. *capri*

**DOI:** 10.1186/1472-6882-12-169

**Published:** 2012-10-02

**Authors:** Amanda V Arjoon, Charlotte V Saylor, Meghan May

**Affiliations:** 1 Department of Biological Sciences, Fisher College of Science and Mathematics, Towson University, Towson, MD, USA; 2 Department of Biological Sciences, Towson University, 8000 York Road, Towson, MD, 21252, USA

## Abstract

**Background:**

Mycoplasmosis is a common infection in human and veterinary medicine, and is associated with chronic inflammation and high morbidity. *Mycoplasma* species are often intrinsically resistant to many conventional antimicrobial therapies, and the resistance patterns of pathogenic mycoplasmas to commonly used medicinal (antimicrobial) plant extracts are currently unknown.

**Methods:**

Aqueous extracts, ethanol extracts, or oils of the targeted plant species and colloidal silver were prepared or purchased. Activity against the wall-less bacterial pathogen *Mycoplasma mycoides* subsp. *capri* was determined and compared to activities measured against *Escherichia coli* and *Bacillus subtilis*. Antimicrobial susceptibility testing was performed by broth microdilution assays. The lethal or inhibitory nature of each extract was determined by subculture into neat growth medium.

**Results:**

Growth of *M*. *mycoides capri*, *E*. *coli*, and *B*. *subtilis* was inhibited by elderberry extract, oregano oil, ethanol extract of oregano leaves, and ethanol extract of goldenseal root. No inhibition was seen with aqueous extract of astragalus or calendula oil. Growth of *M*. *mycoides capri* and *B*. *subtilis* was inhibited by ethanol extract of astragalus, whereas growth of *E*. *coli* was not. Similarly, *M*. *mycoides capri* and *E*. *coli* were inhibited by aqueous extract of thyme, but *B*. *subtilis* was unaffected. Only *B*. *subtilis* was inhibited by colloidal silver. Measured MICs ranged from 0.0003 mg/mL to 3.8 mg/mL. Bacteriostatic and bactericidal effects differed by species and extract.

**Conclusions:**

The atypical pathogen *M*. *mycoides capri* was sensitive to extracts from many medicinal plants commonly used as antimicrobials in states of preparation and concentrations currently available for purchase in the United States and Europe. Variation in bacteriostatic and bactericidal activities between species and extracts indicates that multiple effecter compounds are present in these plant species.

## Background

Atypical bacteria lack or produce aberrant forms of cellular components that characterize most bacterial species. Members of the genus *Mycoplasma* lack cell walls, do not synthesize nucleotides or amino acids, express an unusual form of RNA polymerase, and certain species produce atypical ribosomes [1]. These biological features make mycoplasmas intrinsically resistant to many antibiotics, and successful treatment options are restricted to tetracyclines, macrolides, and fluoroquinolones. *Mycoplasma* infections are a grave concern in veterinary medicine, where they are associated with detrimental and economically burdensome infections in production animals including cattle, goats, swine, chickens, and turkeys [2]. Human mycoplasmosis is associated with numerous clinical manifestations, most notably community acquired pneumonia and nongonococcal urethritis [3,4].

The *in vitro* inhibitory effects of goldenseal (*Hydrastis canadensis*)*,* huang qi (*Astragalus membranaceus*)*,* oregano (*Origanum vulgare*)*,* thyme (*Thymus vulgaris*)*,* elderberry (*Sambucus nigra*)*,* and calendula (*Calendula officinalis*) have been observed and reported for true gram-positive and gram-negative bacteria (Table 1) [5-10]. Their effect on wall-less atypical bacteria such as mycoplasmas is virtually unexplored. A small number of ethnopharmacological studies have explored antimycoplasmal effects of medicinal plants used in Jordan [11] and Nigeria [12] but the impact of botanicals widely used in the United States and Europe on mycoplasmas remains unknown. The lack of classical antimicrobial targets make mycoplasmas intrinsically resistant to many antibiotics [1], and it is unknown if they are similarly resistant to crude and purified extracts from plants commonly used to treat infections in botanical medicine. We examined commercial extracts of the *H. canadensis*, *A. membranaceus*, *O. vulgare*, *T. vulgaris*, *S. nigra*, and *C. officinalis* for efficacy against the small ruminant pathogen *Mycoplasma mycoides* subsp. *capri*. Whole plant extracts and colloidal silver (commercial or freshly prepared) were used at retail concentrations and preparations to better simulate the conditions under which these compounds would be utilized to treat infection *in vivo*. The aim of this study is to determine the efficacy of several commercial and fresh extracts of plants commonly used in botanical medicine (*Hydrastis canadensis, Astragalus membranaceus, Origanum vulgare, Thymus vulgaris, Sambucus nigra, and Calendula officinalis*) against atypical pathogenic bacterial species that lack many common antimicrobial targets, represented here by *M. mycoides capri*. *Escherichia coli* and *Bacillus subtilis* were used as a standard for comparison with eubacterial species in our experimental setting.

**Table 1:** **Plant sources used in this study**^**a**^

**Plant**	**Common name**	**Active compound**	**Chemical family**	**Extract spectrum**	**Reference**
*Hydrastis canadensis*	Goldenseal	Berberine	Alkaloid	G+, G-	[8]
*Astragalus membranaceus*	Astragalus	Polysaccharides (?)	Polysaccharide	Unk	[9]^b^, [13]
*Origanum vulgare*	Oregano	Carvacrol, Thymol	Phenol	G+, G-	[6,14]
*Thymus vulgaris*	Thyme	Carvacrol	Phenol	G+, G-	[10]
*Sambucus nigra*	Elderberry	Tannins, lupeol	Flavonoid, Triterpene	G+, G-	[7,15,16]
*Calendula officinalis*	Calendula	Unk	N/A	Unk	[5]

^a^Abbreviations: G + (Gram positive); G- (Gram negative), Unk (unknown); N/A (not applicable).

^b^Reference is a review article.

## Methods

### Bacterial strains and culture conditions

*Mycoplasma mycoides* subspecies *capri* strain GM12 was cultured in American Type Culture Collection (ATCC) medium 988 (SP-4) containing 20% v/v fetal bovine serum and 1% w/v D-glucose. *Escherichia coli* strain DH5α and *Bacillus subtilis* strain ATCC 6051 (Marburg^T^ strain) were cultured in modified trypticase soy (mT-soy) medium supplemented with 0.002% w/v phenol red, 1% w/v D-glucose, and 1% w/v D-mannitol to ensure colorimetric visualization of growth. All cultures were incubated at 37°C.

### Plant extracts

Commercial preparations (aqueous, ethanol, or oil) of *H. canadensis*, *A. membranaceus*, *O. vulgare*, *T. vulgaris*, *S. nigra*, and *C. officinalis* are described in Table 2. Reported concentrations in mg/mL reflect a w/v measurement of crude plant material to solvent. Aqueous and ethanol extracts of *H. canadensis* and *A. membranaceus* were prepared as previously described [17] (referred to as “fresh” or “freshly prepared”). Briefly, 10 g dried, ground plant material was suspended in 100 mL of molecular-grade water or 95% ethanol. Cellular materials were disrupted by sonication, and suspensions were rapidly shaken on an orbital shaker for 48 hours. Material in suspension was removed by repeated centrifugation at 4,000 RPM followed by vacuum filtration through a 0.45 μM pore nitrocellulose membrane. Fresh and commercial extracts were stored at 4°C.

**Table 2:** **Extract properties**^**a**^

**Extract**	**Manufacturer**	**Concentration (mg/mL)**^**b**^	**Solvent**	**Additional components**^**c**^
Astragalus-Aq	N/A	100	Water	None
Astragalus-ETOH	N/A	100	95% ethanol	None
Astragalus-Com	Gaia Herbs	500	50% ethanol	None
Goldenseal-Aq	N/A	100	Water	None
Goldenseal-ETOH	N/A	100	95% ethanol	None
Goldenseal-Com	Gaia Herbs	10^d^	65% ethanol	None
Elderberry-G	Gaia Herbs	380	Water	C_12_H_22_O_11_, Acerola extract
Elderberry-365	365 Everyday Value	100	Water	C_3_H_8_O_3_, C_6_H_7_KO_2_
Oregano-LE	Gaia Herbs	333	65% ethanol	None
Oregano oil	North American Herb and Spice	1667	Olive oil	None
Thyme	Nature’s Answer	1000	15% ethanol	C_3_H_8_O_3_
Calendula	Eclectic Institute	945	Olive oil	None
Colloidal silver	Source Naturals	0.03	Water	None

^a^Abbreviations: N/A (not applicable/extracts produced for this study); Aq (aqueous extract); ETOH (ethanol extract); Com (commercial extract); LE (leaf extract).

^b^Starting concentrations are those reported for commercial products, and were not normalized to maximize relevance to common use practices.

^c^Role of additional components: C_12_H_22_O_11_ (sucrose)-sweetener; C_3_H_8_O_3_ (glycerol)-texture, sweetener; C_6_H_7_KO_2_ (potassium sorbate)-antifungal preservative; Acerola extract-ascorbic acid supplementation, anti-inflammatory properties, additional antimicrobial properties^28^.

^d^Concentration reported by the manufacturer refers to total alkaloids rather than plant material.

### Antimicrobial susceptibility testing

Stock cultures of *M. mycoides capri*, *E.coli*, and *B. subtilis* were diluted to 0.5 McFarland standard in sterile SP-4 or mT-soy medium. Minimum inhibitory concentrations (MIC) were determined by broth microdilution as previously described [18] using SP-4 or mT-soy as the diluents for serial 10-fold dilutions of extracts. 10% solutions of molecular grade water, 95% ethanol (*i.e.*, 9.5% final concentration), and olive oil were inoculated with each species to control for the effect of plant extract solvents. Starting concentrations of each extract ranged from 1667 to 0.03 mg/mL and are reported in Table 2. Broth microdilution assays were incubated for 18 hours at 37°C. Bacterial growth was noted by acidification of the medium observed colorimetrically by shifting of the pH indicator phenol red. The bacteriostatic versus bactericidal nature of the extracts was determined by subculture from wells containing inhibitory dilutions into neat SP-4 or mT-soy medium (broth and agar) at 37°C. Bactericidal activity of an extract was noted after incubation for 7 days and an absence of colonies on agar plates. Minimum bacteriostatic concentrations (MBCs) were recorded. All susceptibility tests were performed three distinct times.

## Results and discussion

The efficacy of commercial and fresh extracts of plants commonly used in botanical medicine to treat infection (*Hydrastis canadensis, Astragalus membranaceus, Oreganum vulgare, Thymus vulgaris, Sambucus nigra, and Calendula officinalis*) and colloidal silver against an atypical pathogenic bacterial species that lacks many common antimicrobial targets was examined. Representative species were selected for analysis based on precedent in antimicrobial assessments of botanical extracts (gram negative species *E. coli* and gram positive species *B. subtilis*) or *in vitro* growth characteristics (atypical species *M. mycoides capri*) [19,20]. Mycoplasmas are notoriously fastidious, but *M. mycoides capri* grows well in axenic culture and thus is ideal for *in vitro* analysis. Microdilution was selected over disk-diffusion because several recent studies have reported greater experimental sensitivity with broth microdilution [21,22]. Intraspecies diversity in resistance to many conventional antibiotics has been noted for *M. mycoides capri* and may also exist for the included antimicrobial extracts [23,24], therefore the use of a single strain represents a limitation of this work. Future studies including numerous strains are needed prior to recommending a standard concentration for treatment with extracts; however, this study represents an essential first step in establishing a baseline expectation for susceptibility using strain GM12.

We found a wide range of resistance and sensitivities with the compounds tested, two of which conflicted with previous findings (Table 3) indicating higher sensitivity of gram positive bacteria to goldenseal alkaloids (analogous to our commercial goldenseal) than of *E. coli*[25], and sensitivity of *E. coli* to colloidal silver by qualitative methods [26]. Growth of *M. mycoides capri*, *E. coli*, and *B. subtilis* was inhibited by all tested elderberry extracts, oregano oil, ethanol extract of oregano leaves, and fresh ethanol extract of goldenseal root. No inhibition was seen with aqueous extract of astragalus or calendula oil. Growth of *M. mycoides capri* and *B. subtilis* was inhibited by fresh ethanol extract of astragalus, whereas growth of *E. coli* was not. Similarly, *M. mycoides capri* and *E. coli* were inhibited by a commercial ethanol extract of thyme, but *B. subtilis* was unaffected. Only *B. subtilis* was inhibited by colloidal silver, and only *M. mycoides capri* was inhibited by fresh aqueous extract of goldenseal root. No inhibition was observed for any species with the extract solvents (*i.e.*, molecular grade water, 95% ethanol, or olive oil).

**Table 3:** **Minimum inhibitory and bacteriostatic concentrations**^**a**^

**Extract**	***E. coli***		***B. subtilis***		***M. mycoides capri***	
	**MIC (mg/mL)**	**MBC**^**b**^**(mg/mL)**	**MIC (mg/mL)**	**MBC**^**b**^**(mg/mL)**	**MIC (mg/mL)**	**MBC**^**b**^**(mg/mL)**
Astragalus-Aq	>10	N/A	>10	N/A	>10	N/A
Astragalus-ETOH	>10	N/A	**0.001**	**0.001**	**1.0**	**BC**
Astragalus-Com	>50	N/A	>50	N/A	>50	N/A
Goldenseal-Aq	>10	N/A	>10	N/A	**0.05**	**0.5**
Goldenseal-ETOH	**1.0**	**1.0**	**1.0**	**BC**	**1.0**	**1.0**
Goldenseal-Com^c^	**0.1**	**BC**	>1	N/A	>1	N/A
Elderberry-G	**0.0038**	**0.0038**	**3.8**	**3.8**	**0.38**	**0.38**
Elderberry-365	**0.1**	**BC**	>10	N/A	**0.001**	**BC**
Oregano-LE	**0.033**	**BC**	**0.00033**	**BC**	**0.033**	**BC**
Oregano oil	**0.167**	**BC**	**0.0167**	**BC**	**0.092**	**0.167**
Thyme	**1.0**	**BC**	>100	N/A	**0.5**	**BC**
Calendula	>95	N/A	>95	N/A	>95	N/A
Colloidal silver	>0.003	N/A	**0.0003**	**0.0003**	>0.003	N/A
Water	NI	N/A	NI	N/A	NI	N/A
95 % Ethanol	NI	N/A	NI	N/A	NI	N/A
Olive oil	NI	N/A	NI	N/A	NI	N/A

^a^N = 3 replicates; MIC/MBC concentrations reflect mean values across trials. Abbreviations: N/A (not applicable); BC (bactericidal); NI (no inhibition by solvents at the lowest dilution [1:10]). Efficacious compounds with a measureable inhibitory concentration or bactericidal activity are bolded.

^b^Columns marked N/A or BC indicate that MBCs could not be determined due to lack of inhibition (N/A) or bactericidal activity (BC).

^c^Concentration reflects total alkaloid content.

While inhibition of *B. subtilis* and *M. mycoides capri* was observed for the fresh astragalus ethanol extract, the commercial ethanol extract and the fresh aqueous extract were ineffective. Our data suggest that the antimicrobial component of *A. membranaceus* is not water-soluble, and that the commercial preparation tested does not retain the component. While we did not observe any inhibition with calendula oil, other studies have documented its positive contribution to wound healing [27-30]. Calendula is associated with lymphocyte activation [15], angiogenesis [30], and alleviation of irritations [31], so it is plausible that its role in healing is due primarily to immune modulation and stimulation of tissue repair rather than antimicrobial activity.

The MICs of compounds that exhibited growth inhibition are presented in Table 3. Measured MICs ranged from 0.0003 mg/mL to 3.8 mg/mL. Commercial ethanol extracts of astragalus differed in their measured MICs from our laboratory-prepared ethanol extracts for *B. subtilis* and *M. mycoides*. Commercial ethanol extracts of goldenseal root differed from our laboratory-prepared ethanol extracts for all three species tested (Table 3). Similarly, two distinct brands of elderberry extract (Gaia Herbs and 365 Everyday Value) had measurable differences in MIC for all three species (Table 3). Finally, whole oregano oil and ethanol extract of oregano leaf showed measurably different MICs for all three organisms (Table 3). The subculture of inhibited wells into neat SP-4 or mT-soy medium indicated a range of bacteriostatic and bactericidal effects that differed by species and extract (Table 4). Aqueous extract of goldenseal and one preparation of elderberry extract (Gaia Herbs) were uniformly bacteriostatic across inhibited species, and oregano oil, the second preparation of elderberry extract (365 Everyday Value), and thyme extract were uniformly bactericidal across inhibited species. The remaining extracts were static for certain species and lethal for others (Table 4).

**Table 4:** **Post-inhibition analysis**^**a**^

**Compound**	***E. coli***	***B. subtilis***	***M. mycoides capri***
Astragalus-Aq	resistant	resistant	resistant
Astragalus- ETOH	resistant	bacteriostatic	bactericidal
Astragalus-com	resistant	resistant	resistant
Goldenseal-Aq	resistant	resistant	bacteriostatic
Goldenseal-ETOH	bacteriostatic	bactericidal	bacteriostatic
Goldenseal-com	bactericidal	resistant	resistant
Elderberry (365)	bactericidal	resistant	bactericidal
Elderberry (G)	bacteriostatic	bacteriostatic	bacteriostatic
Oregano leaf	bactericidal	bactericidal	bactericidal
Oregano oil	bactericidal	bactericidal	bacteriostatic
Colloidal Silver	resistant	bactericidal	bactericidal
Thyme	bactericidal	resistant	bactericidal
Calendula	resistant	resistant	resistant

^a^Definition of terms: **“resistant”**-species was resistant to the compound; no analysis performed; **“bacteriostatic”**: compound was shown to be bacteriostatic by growth upon subculture; **“bactericidal”**: compound was shown to be bactericidal by lack of growth upon subculture. N = 2 replicates.

*S. nigra* (elderberry) has been traditionally used to treat influenza, but a small number of additional studies show that this compound inhibits growth of gram positive and gram negative bacteria [25,32]. Inhibition was observed for both formulations of elderberry used in this study, but direct comparisons are difficult to make due to additional components in the formulation. The Gaia Herbs formulation contained acerola extract, which has also been reported to have antimicrobial activity [14]. However, Motohashi *et al.* reported the acerola was ineffective against all gram negative bacteria tested, while gram positive bacteria were sensitive. We found the MIC of the gram negative *E. coli* was 100-fold lower than the MICs of the gram positive *B. subtilis* and the atypical *M. mycoides capri* for this formulation of elderberry, indicating that acerola was not a major component of the observed inhibition. The antifungal and antibacterial preservative potassium sorbate (C_6_H_7_KO_2_) was added to the 365 Everyday Value formulation of elderberry. The impact of C_6_H_7_KO_2_ was apparent from the post-inhibition analysis, where the 365 Everyday Value formulation was found to be bactericidal and the Gaia Herbs preparation was found to be bacteriostatic. The antibacterial effect of C_6_H_7_KO_2_ is bactericidal [33], and so the presence of this preservative likely contributes to the analytic discrepancy and the inhibitory effect of the 365 Everyday Value preparation. While inhibition was observed for *E. coli* and *M. mycoides capri* with this preparation, the MIC was 2-fold higher for *E. coli*. In contrast, the MIC was 100-fold lower for *M. mycoides capri*, potentially indicating a greater sensitivity of atypical bacteria to the preservative C_6_H_7_KO_2_. Susceptibility testing using C_6_H_7_KO_2_ alone was not performed, but would confirm this indication. In further contrast, *B. subtilis* was resistant to the 365 formulation of elderberry at concentrations above the MIC measured for the Gaia Herbs formulation indicating that either 1.) the differential in MIC for *B. subtilis* can be attributed to the anti-gram positive effects of acerola; 2.) the efficacy of elderberry’s antimicrobial components is sensitive to shelf life; or 3.) elderberry and acerola act synergistically against *B. subtilis* in a way that elderberry and C_6_H_7_KO_2_ do not. In any case, it is clear that there are marked differences in *in vitro* efficacy between these formulations for all three representative types of bacteria.

Comparisons between commercial and freshly prepared ethanol extracts of astragalus and goldenseal root lend support to the notion of shelf life being an underappreciated problem, though differences in processing or preparation have not been accounted for. Fresh astragalus extract inhibited growth of *B. subtilis* and *M. mycoides capri*, while the commercial extract was ineffective against all three species. Similarly, the fresh ethanol extract of goldenseal root inhibited the growth of all three species, while *B. subtilis* and *M. mycoides capri* were completely resistant to the commercial extract. Curiously, *E. coli* had a 10-fold lower MIC for the commercial extract, but this is likely due to the reported concentration reflecting total alkaloid content rather than the dry weight of the whole root as was the case for the fresh ethanol extract. The alkaloid berberine is recognized as the major antibacterial component of goldenseal root [8], indicating that the commercial extract should intrinsically have a lower measured MIC than the fresh ethanol extract. Room-temperature storage of commercial and freshly prepared extracts with periodic measurements of the MICs for each would appropriately assess the scale of this problem.

Our data collected from oregano preparations and our post-inhibition analysis support findings from previous studies [17] indicating that commercial extracts of medicinal plants contain multiple antimicrobial compounds. Ethanol extract from oregano leaves had lower MICs than oil emulsion of whole oregano for all three species, and this difference was remarkably pronounced for *B. subtilis* and *M. mycoides capri*. This difference likely stems from 1.) differential solubility of antibacterial compounds in oil versus ethanol, 2.) components present in the stem and root acting antagonistically to the effect exhibited by components in the leaves, or both. Lambert *et al.* reported that multiple antibacterial compounds are present in oregano plants [34], but this work was later contradicted by Peñalver *et al.* who associated the activity exclusively with the phenolic compound carvacrol [35]. However, there is greater evidence for the presence of multiple compounds in the form of findings showing a greater antimicrobial effect of oregano oil than of purified carvacrol [36]. Similarly, our findings of an extract being bacteriostatic for one species and bactericidal for another (as was the case for oregano leaf extract and fresh ethanol extracts of goldenseal root and astragalus) suggests multiple components within the same extracts exhorting different biological effects. This likely explains the unexpected finding of a wide range of sensitivity by *M. mycoides capri* when intrinsic antimicrobial resistance is predictable [1].

Our data indicate that commercial preparations of medicinal plants are predictably variable in their MIC and antibacterial spectrum. Because most are commercial extracts, however, the concentrations used are relevant to common usage scenarios despite the extracts having potential to be inhibitory at higher concentrations. Perhaps surprisingly the wall-less, atypical species *M. mycoides capri* was not limited in its sensitivity profile as compared to the eubacterial species *E. coli* and *B. subtilis*.

## Conclusion

Interest in botanical medicine has led to widespread availability of antimicrobial plant extracts in the United States. We found a wide range of resistance and sensitivities with the compounds tested, the majority of which were consistent with previous findings for eubacteria. In the case of thyme ethanol extract, our findings complement a previous report describing the effects of an aqueous extract [37]. The *in vitro* efficacy of these extracts at their available concentrations on atypical bacteria is vastly understudied, and our study represents an important first step in establishing the efficacy of these compounds as botanical treatments for mycoplasmosis. Further exploration into the toxicity, bioavailability, pharmacokinetics, and ultimately the *in vivo* efficacy against mycoplasmosis of these extracts will provide evidence for their potential use as treatments or for the disinfection of agricultural or clinical fomites such as milking equipment. Based on the measured antimicrobial effects of the medicinal plants we studied, common commercial preparations of astragalus (ethanol extracts), goldenseal root (aqueous or ethanol extracts), elderberry, oregano (leaf extracts or essential oil), and thyme have potential to be primary or ancillary treatments for mycoplasmosis, and further study *in vivo* is required.

## Competing interests

The authors declare that they have no competing interests

## Authors’ contributions

AVA and MM carried out experimental procedures. MM and CVS designed the study, drafted, and revised the manuscript. Authors read and approved the final manuscript.

## Pre-publication history

The pre-publication history for this paper can be accessed here:

http://www.biomedcentral.com/1472-6882/12/169/prepub
